# Exploring Social-Ecological Pathways From Sexual Identity to Sleep Among Chinese Women: Structural Equation Modeling Analysis

**DOI:** 10.2196/53549

**Published:** 2025-01-21

**Authors:** Chanchan Wu, Pui Hing Chau, Edmond Pui Hang Choi

**Affiliations:** 1 School of Nursing, Li Ka Shing Faculty of Medicine, The University of Hong Kong, 5/F, Academic Building, 3 Sassoon Road, Pok Fu Lam, Hong Kong, China (Hong Kong), 852 39176972

**Keywords:** sleep, social support, sexual minority women, social-ecological model, quality of life, structural equation model, Chinese women, China, women, structural equation modeling analysis, sleep quality, sexual identity, survey, heterosexual, cisgender

## Abstract

**Background:**

Women and sexual minority individuals have been found to be at higher risk for experiencing poor sleep health compared to their counterparts. However, research on the sleep health of sexual minority women (SMW) is lacking in China.

**Objective:**

This study aimed to examine sleep quality and social support for Chinese women with varied sexual identities, and then investigate the in-depth relationships between sexual identity and sleep.

**Methods:**

This was a cross-sectional web-based survey. All participants completed a structured questionnaire containing a set of sociodemographic items referring to the social-ecological model of sleep health, the Pittsburgh Sleep Quality Index, the Social Support Rating Scale, and social relationships and environment domains of the World Health Organization Quality of Life-abbreviated short version. Pearson correlation coefficients were used to examine the relationship between sleep quality and social support as well as the two domains of quality of life. Structural equation modeling analysis was used to explore the social-ecological relationships.

**Results:**

A total of 250 cisgender heterosexual women (CHW) and 259 SMW were recruited from July to September 2021. A total of 241 (47.3%) women experienced poor sleep quality and the rate was significantly higher in SMW than in CHW (55.2% vs 39.2%, *P*<.001). Around one-fifth of SMW reported low levels of social support, which was significantly higher than that of CHW (21.6% vs 5.6%, *P*<.001). Pearson correlations showed that overall sleep quality was significantly negatively associated with social support with weak correlations (*r=*−0.26, *P*<.001). The final structural equation modeling analysis with satisfactory fit indices identified 6 social-ecological pathways, showing that alcohol use, objective support, utilization of support, and perceived social relationship and environment quality of life played important roles in the sleep quality of individuals from their sexual identity.

**Conclusions:**

SMW experienced poorer sleep quality compared to CHW. Further research is recommended to address the modifiable factors affecting sleep and then implement tailored sleep improvement programs.

## Introduction

Sleep is a crucial element that significantly impacts health across all populations. As a modifiable behavior, sleep is strongly linked to health and well-being [1], and abnormal sleep has been proven to be signiﬁcantly associated with an extensive range of adverse health-related outcomes [2,3]. Consequently, sleep health is increasingly recognized as a public health issue that concerns everyone [4,5]. Sleep quality is widely used to evaluate sleep health and includes both objective and subjective aspects of sleep [6]. Many studies have shown that women generally experience poorer sleep quality than age-matched men, possibly due to the influence of sex steroids over sleep [7,8]. Although research on sleep health among vulnerable populations has gradually increased, most studies have been limited to racial or ethnic minorities [7,9-12], and knowledge of sleep health among sexual minority populations is relatively scarce [13].

To date, only 3 reviews have been published on the sleep health of sexual minorities, with all being narrative reviews that contain a very limited number of studies [14-16], reflecting the fact that relevant research is still in its infancy. Overall, these reviews suggest that sleep health constitutes an unmet health need for sexual minorities, and sleep health disparities related to sexual identity have been widely documented. Consistently, existing studies have recognized that sexual minority individuals experience significantly worse sleep quality than their cisgender and heterosexual peers. Furthermore, women and sexual minority individuals reported more sleep difﬁculties than did men or heterosexual participants [17], and sexual minority women (SMW) were more likely to report a higher prevalence of poor sleep quality than heterosexual women regardless of race/ethnicity [18-20], especially those in less supportive environments [21]. Additionally, SMW were more vulnerable to experiencing sleep disturbances than both sexual minority and heterosexual men [22], indicating that the sleep health of SMW deserves more attention.

The current evidence on sleep health among sexual minority Chinese is very limited; it remains an understudied area of research. So far, there are only 3 relevant studies, 2 of which are based on the analysis of data from nationwide school-based surveys of adolescents [23,24] and college students [25], indicating that sexual minority status was significantly associated with poor sleep quality. Notably, there is only 1 study that specifically investigated the sleep and discrimination experienced by lesbian, gay, and bisexual individuals in Hong Kong [26], and the results showed that discrimination experienced was associated with greater sleep disturbance, which in turn led to poorer physical and mental health conditions.

Health-related research on sexual minority Chinese also exhibits significant gender disparities, manifested by a substantially higher focus on the male sexual minority population than on female groups [27]. A recently published scoping review that mapped all the scientific literature and gray reports on the health needs of women with same-sex attraction in mainland China determined that Chinese sexual minority women have multiple unmet health needs [28], such as substance abuse, concerning mental health, and sexual and reproductive health. However, little is known about their sleep health, which is a crucial health issue that deserves more attention.

Research on factors affecting sleep has received more attention in recent years [29,30]. Grandner et al [31,32] initially proposed the social-ecological model of sleep health that integrated possible determinants of sleep and sleep-induced health outcomes from a global perspective. This model has been applied to studies in different populations [9,33,34] but has not yet been applied to sexual minority populations. Regarding the determinants of sleep, this model considers from a socio-ecological perspective that sleep may be determined by multiple causes at 3 levels, including the individual level, social level, and societal level.

Individual-level factors contain all aspects directly related to individuals’ sleep such as genetics and sleep-related behaviors. A growing body of literature suggests that alcohol use has detrimental effects on sleep [35] by reducing sleep quality [36]. Meanwhile, there is also comprehensive evidence that the pooled association between alcohol use and sleep disorders was not significant [37], suggesting that the relationship between alcohol use and sleep needs further investigation. Similarly, smoking has been identified as another sleep determinant [38], with smokers being more vulnerable to poorer sleep quality [39]. In China, a review targeting sexual minority women concluded that the prevalence of smoking and alcohol use among minority women was much higher than among general women [28]. Therefore, alcohol use and smoking status need to be considered along with sexual identity when exploring individual-level factors that affect sleep quality.

Social-level factors influencing sleep could be socioeconomic status, ethnicity, and social support. Ethnic minorities and individuals from disadvantaged economic backgrounds generally report lower sleep quality [12,13]. In addition, a review of sleep research concluded that financial hardship is associated with poor sleep health in the general population [16]. Notably, many studies have confirmed that social support is a key factor affecting sleep [40,41], with greater social support associated with better sleep outcomes [42-45], while having strained relationships was linked to more troubled sleep [46]. Compared with heterosexual populations, social support has unique functions in sexual minority individuals, but they reported receiving less support [47]. Therefore, when exploring social-level factors affecting sleep quality, conditions such as ethnicity, education, employment, and economic status need to be considered in addition to social support.

Regarding societal-level factors affecting sleep, geography, physical environment, and other aspects of the environment could be grouped into this category [32], and environmental factors may positively or negatively impact sleep [48]. A cohort study also found a link between declining social relationship quality and poor sleep quality [49]. The societal-level factors are closely linked to everyone’s livelihood, and therefore it is worth exploring whether individuals are local residents or new migrants to their current residence and whether they are cohabitating with others. The abovementioned factors involved in the social-ecological model might have direct or indirect impacts on sleep, but the associations among these factors have not been fully studied.

Despite the evident vulnerability of women and sexual minorities to poor sleep, there has been little research on their sleep health. Meanwhile, in China, research on the sleep health of sexual minorities, although gradually receiving attention in recent years, is still very limited, and there is currently no research specifically on the sleep of sexual minority women. To help eliminate inequalities in sleep health, a more diverse and inclusive sample, including women of different sexual identities, is greatly needed. Therefore, this study aimed to examine the sleep quality and social support in Chinese adult women, compare them between SMW and cisgender heterosexual women (CHW), then investigate the in-depth relationships between sexual identity and sleep using structural equation modeling (SEM) in the Chinese context.

## Methods

### Participants and Recruitment

This was a web-based cross-sectional study. The Strengthening the Reporting of Observational Studies in Epidemiology (STROBE) checklist [50] was used for reporting the study findings. Eligible participants were Chinese women who: (1) were at least 18 years old, (2) self-identiﬁed as female or were assigned female gender at birth, and (3) were able to read and understand Mandarin Chinese.

Generally, the minimum sample size for SEM is 200 [51,52], and the sample size needs to be more than 25 times the number of parameters as a rule of thumb [53]. In this study, based on the hypothesized model, it is evident that 10 variables are expected to be entered into the SEM model validation, thus the minimum sample size is 250.

Convenience sampling and respondent-driven sampling methods were used to recruit this relatively hidden population [54,55], by releasing the study poster via 4 popular nongovernmental organizations and encouraging respondents to help recruit potential peers through their network of connections. Details regarding the recruitment procedure have been previously reported [56].

In this study, 524 questionnaires were collected between July and September 2021, of which 15 were excluded for being cisgender men, adolescents, or invalid data. Finally, a total of 509 (97.1%) adult women were enrolled in the study with no missing data.

### Ethical Considerations

This study was approved by the Human Research Ethics Committee of the University of Hong Kong (reference number EA210325) on July 8, 2021. Electronic written consent was obtained for each study participant. Data were collected using an online survey platform (Wenjuanxing), and no compensation was provided. All information collected during the study was kept anonymous and strictly confidential.

### Measures

#### Pittsburgh Sleep Quality Index

The Pittsburgh Sleep Quality Index (PSQI) is a commonly used measure of individuals’ sleep quality [14,57]. It includes 19 self-rated items, and they generate 7 component scores with an average weight of 0‐3 points. The Chinese version of the PSQI has been previously validated [58,59] and was used in this study. Specifically, the seven components are: (1) subjective sleep quality, (2) sleep latency, (3) sleep duration, (4) habitual sleep efficiency, (5) sleep disturbances, (6) sleeping medication use, and (7) daytime dysfunction. The sum of the 7 component scores yields 1 global score, ranging from 0 to 21 points, and higher scores represent poorer sleep quality. A global PSQI score >5 indicates poor sleep quality [6,57].

#### Social Support Rating Scale

The Social Support Rating Scale (SSRS) is a 10-item scale specially designed for the Chinese population with sound reliability and validity [60]. It includes three dimensions of social support: (1) subjective support, (2) utilization of support, and (3) objective support. The full-scale score and dimension scores are the sum of the scores of each item, with higher scores indicating higher levels of social support [61,62]. In addition, a total score of 22 or below could be classified as low level, 23‐44 as medium level, and 45‐66 as a high level of social support [63].

#### World Health Organization Quality of Life – Abbreviated Short Version

The World Health Organization Quality of Life – Abbreviated Short Version (WHOQOL-BREF) is a generic quality of life (Qol) measure comprising 4 domains, and the psychometric properties of its Chinese version have been conﬁrmed [64-66]. According to the social-ecological model of sleep [32], social relationships and environment could be considered as the societal-level factors affecting sleep, but there is little evidence of associations between sleep and these factors. Thus, this study used the social relationship domain and environment domain of the WHOQOL-BREF as societal-level factors of sleep for investigation. Each domain score can be transformed into a score ranging from 4 to 20, with higher scores representing better Qol [64,67].

#### Sociodemographic Information

All participants completed a set of sociodemographic items that were widely reported in previous studies referring to the social-ecological model of sleep health [31,32], including individual-level factors (sexual identity, age, smoking status, alcohol use status, drug use status), social-level factors (ethnicity, education, employment, income, number of friends, social support, relationship status, bed-sharing situation), and societal-level factors (local resident or not, duration in current residence, cohabitation situation, social relationship and environment Qol).

### Statistical Analysis

Descriptive statistics were reported on participants’ demographic characteristics, sleep quality, and social support. Independent *t* tests and *χ*^2^ tests were conducted for comparisons between SMW and CHW. Cohen *d* effect size was calculated [68].

#### Social-Ecological Factors Identification

Pearson correlation coefficients were used to examine the relationship between sleep quality and social support as well as the 2 domains of quality of life, and the correlations were defined as strong (≥0.5), moderate (≥0.3 and <0.5), or weak (<0.3) [68]. Then, independent *t* tests, 1-way ANOVA, and multiple linear regressions were performed to identify factors significantly associated with sleep quality.

#### Structural Equation Modeling Analysis

**Figure 1.**
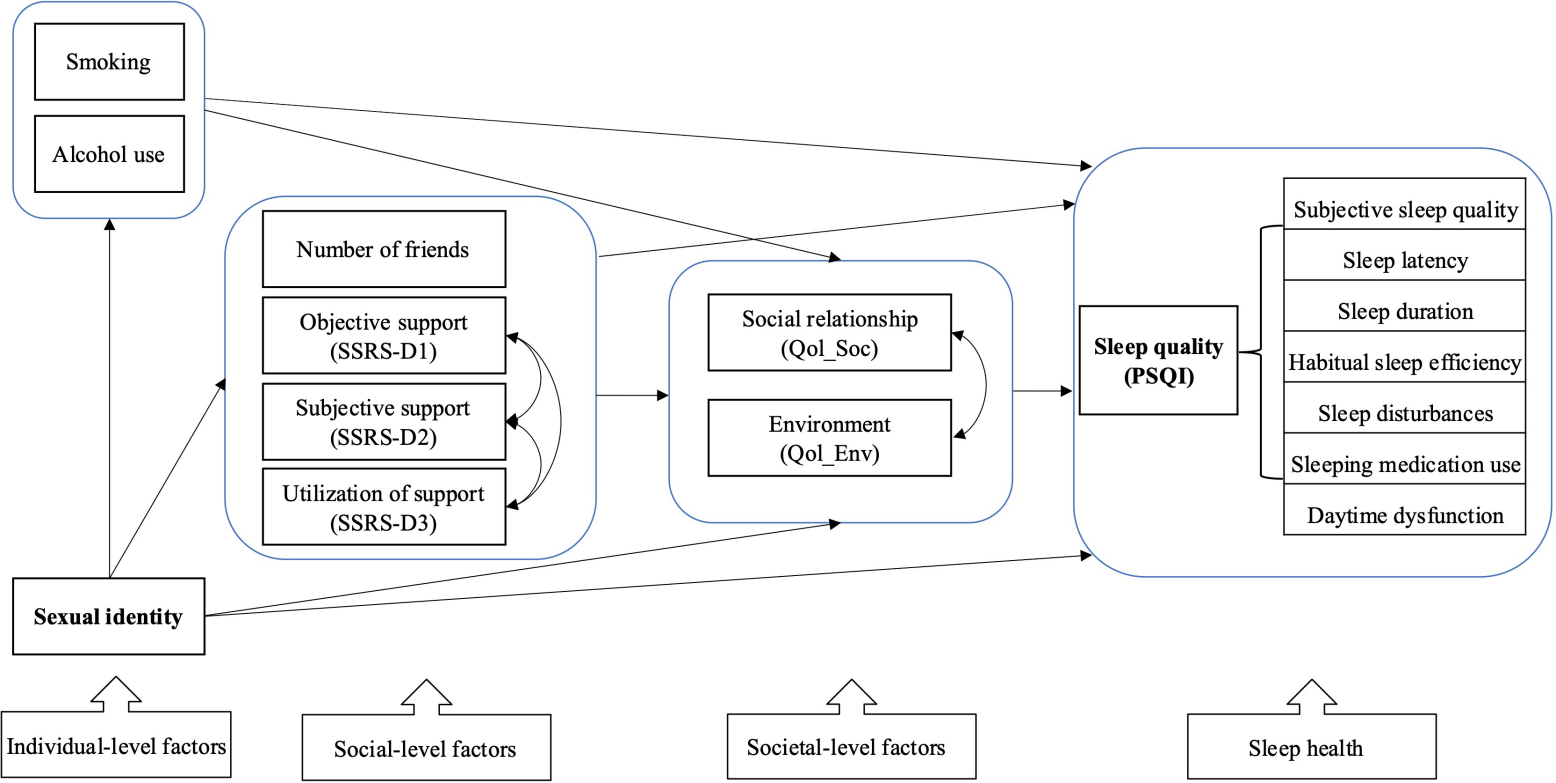
Hypothesized model for socioecological inﬂuences on sleep (based on the social-ecological model of sleep). PSQI: Pittsburgh Sleep Quality Index; Qol: Quality of life; Qol_Env: Environment domain of Qol; Qol_Soc: Social relationship of Qol; SSRS: Social Support Rating Scale; SSRS-D1: objective support; SSRS-D2: subjective support; SSRS-D3: utilization of support.

Based on the factors identified previously (which will be further detailed in the Results section), with reference to the socio-ecological model of sleep, the following factors with significant coefficients were simultaneously entered into the SEM analysis, including sexual identity (binary), alcohol use (binary), and smoking (binary) (identified as individual-level factors); the number of friends (categorical) and social support (continuous) (identified as social-level factors); and social relationship Qol (continuous) and environment Qol (continuous) (identified as societal-level factors). Sleep quality was treated as the latent construct using the 7 domains of the PSQI measure. The hypothesized model for socioecological inﬂuences on sleep based on the social-ecological model of sleep is shown in Figure 1.

All identified variables and the 7 components of sleep quality were included in the SEM model to test the hypothesized relationships among the previously listed variables. Standardized coefficients (β), standard errors, and associated *P* values are reported for all paths of the final model. Model goodness of fit was evaluated using chi-square (*χ*^2^), root mean square error of approximation (RMSEA), comparative fit index (CFI), Tucker-Lewis index (TLI), and standardized root mean square residual (SRMR). The model fit was considered adequate [69,70] when RMSEA (90% CI) ≤0.08, CFI ≥0.90, TLI ≥0.90, and SRMR ≤0.05.

Data were analyzed using SPSS version 28.0 (IBM Corp), and the SEM analysis was performed using Mplus 8.6 software [71]. All signiﬁcance tests were 2-tailed, and findings with *P* values <.05 were considered statistically signiﬁcant.

## Results

### Participant Characteristics

Table 1 presents the sociodemographic characteristics of the 509 women. Approximately half of the participants (259/509, 50.9%) were SMW, and 250 (49.1%) were CHW. The mean age of the overall sample was 25.57 years (SD 5.77), ranging from 18 to 56 years old. Details regarding the sexual orientation and gender identity of participants have been previously reported [56]. Notably, SMW had higher rates of substance use than CHW, and the rates of smoking and alcohol use showed significant differences (27.4% vs 4%, *P*<.001; 73.7% vs 56%, *P*<.001; respectively). Nine participants in total (1.8%) had a history of recreational drug use (4 used cannabis and 5 used methamphetamine), 7 of whom were SMW.

**Table 1.** Sociodemographic characteristics of the study sample with comparisons (N=509).

Characteristics	Overall (N=509)	SMW^a^ (n=259)	CHW^b^ (n=250)
**Smoking, n (%);** ***P*****<.001**^c^			
Never smoked or have quit smoking	428 (84.1)	188 (72.6)	240 (96)
Current smoker	81 (15.9)	71 (27.4)	10 (4)
Seldom (<1 time/week)^d^	48 (59.3)	41 (57.7)	7 (70)
Usually (1-7 times/week)^d^	9 (11.1)	9 (12.7)	0
Almost smoke every day^d^	24 (29.6)	21 (29.6)	3 (30)
**Alcohol use, n (%);** ***P*****<.001**			
Never drank or have quit	178 (35)	68 (26.3)	110 (44)
Current alcohol user	331 (65)	191 (73.7)	140 (56)
Seldom (<1 time/week)^e^	220 (43.2)	111 (42.9)	109 (43.6)
Occasionally (2‐4 times/month)^e^	88 (17.3)	64 (24.7)	24 (9.6)
Usually (2‐4 times/week)^e^	17 (3.3)	13 (5)	4 (1.6)
Almost use alcohol every day^e^	6 (1.2)	3 (1.2)	3 (1.2)
**Drug use, n (%);** ***P*****=.10**			
Never used before	500 (98.2)	252 (97.3)	248 (99.2)
Have used drugs before	9 (1.8)	7 (2.7)	2 (0.8)
**Ethnicity, n (%);** ***P*****=.12**			
Han people	466 (91.6)	242 (93.4)	224 (89.6)
Others (Muslim, etc)	43 (8.4)	17 (6.6)	26 (10.4)
**Education, n (%);** ***P*****<.001**			
High school and below	33 (6.5)	21 (8.1)	12 (4.8)
College/bachelor	284 (55.8)	167 (64.5)	117 (46.8)
Graduate degree and above	192 (37.7)	71 (27.4)	121 (48.4)
**Monthly income (Chinese yuan)^f^, n (%);** ***P*****<.001**			
≤1000	163 (32)	88 (54)	75 (46)
1001‐3000	79 (15.5)	51 (64.6)	28 (35.4)
3001‐5000	40 (7.9)	23 (57.5)	17 (42.5)
5001‐7000	33 (6.5)	17 (51.5)	16 (48.5)
7001‐9000	45 (8.8)	24 (53.3)	21 (46.7)
9001‐11,000	38 (7.5)	16 (42.1)	22 (57.9)
More than 11,000	111 (21.8)	40 (36)	71 (64)
**Relationship, n (%);** ***P*****<.001**			
Have a steady partner	251 (49.3)	115 (44.4)	136 (54.4)
Have no steady partner(s)	258 (50.7)	144 (55.6)	114 (45.6)
**Bed sharing status, n (%);** ***P=*****.12**			
Separate bed in separate room	293 (57.6)	160 (61.8)	133 (53.2)
Separate bed in shared room	76 (14.9)	37 (14.3)	39 (15.6)
Sharing same bed with partner	140 (27.5)	62 (23.9)	78 (31.2)
**Local resident or not, n (%);** ***P=*****.52**			
Local resident	181 (35.6)	96 (37.1)	85 (34)
Nonlocal resident (migrant)	328 (64.4)	163 (62.9)	165 (66)
**Duration in current residence, n (%);** ***P=*****.59**			
<3 months	50 (9.8)	27 (10.4)	23 (9.2)
3‐6 months	17 (3.3)	11 (4.2)	6 (2.4)
7‐12 months	33 (6.5)	15 (5.8)	18 (7.2)
>1 year	409 (80.4)	206 (79.5)	203 (81.2)
**Cohabitation status, n (%); ** ***P*** **<.001**			
Live alone	114 (22.4)	60 (23.2)	54 (21.6)
Live with same-sex partner	41 (8.1)	34 (13.1)	7 (2.8)
Live with opposite-sex partner	75 (14.7)	18 (7)	57 (22.8)
Live with friends	91 (17.9)	42 (16.2)	49 (19.6)
Live with family	162 (31.8)	93 (35.9)	69 (27.6)
Other	26 (5.1)	12 (4.6)	14 (5.6)
**Quality of life, mean (SD); ** ***P*** **<.001**			
Social relationship domain	13.62 (3.07)	13.02 (3.35)	14.23 (2.61)
Environment domain	13.74 (2.68)	13.32 (2.90)	14.18 (2.35)

a SMW: sexual minority women. The percentages for this column were all calculated with 259 SMW as the denominator.

b CHW: cisgender heterosexual women. The percentages for this column were all calculated with 250 CHW as the denominator.

c All *P* values were reported by conducting comparisons performing *χ*2 tests or independent *t* tests.

d The denominator for this row is the number of current smokers (n=81).

e The denominator for this row is the number of current alcohol users (n=331).

f 1 CNY=US $0.136612.

### Sleep Quality and Social Support

A total of 241 (47.3%) women experienced poor sleep quality and the rate was significantly higher in SMW than in CHW (55.2% vs 39.2%, *P*<.001). Similarly, independent *t* tests showed that SMW experienced significantly worse overall sleep quality than CHW (*P*<.001). There were statistically significant differences in subjective sleep quality, sleep latency, habitual sleep efficiency, sleep disturbances, and daytime dysfunction between CHW and SMW (Table 2). In addition, 81.5% (415/509) of all participants reported medium levels of social support. Around one-fifth of SMW reported low levels of social support, which was significantly higher than that of CHW (21.6% vs 5.6%, *P*<.001). Table 2 shows that SMW reported significantly lower support in all aspects than CHW (*P*<.01).

**Table 2.** Sleep quality and social support of the study sample with comparisons (N=509).

	Overall (N=509)	SMW^a^ (n=259)	CHW^b^ (n=250)	*P* value	Chi-square (*df*) or Cohen *d*
**Sleep quality (PSQI** ^**c**^ **), n (%)**					
Good (total score ≤5)	268 (52.7)	116 (44.8)	152 (60.8)	<.001	13.08 (1)
Poor (total score >5)	241 (47.3)	143 (55.2)	98 (39.2)		
**Sleep quality (PSQI), mean (SD)**					
Subjective sleep quality	1.13 (0.74)	1.22 (0.74)	1.04 (0.72)	.005	0.25
Sleep latency	1.24 (0.98)	1.33 (1.01)	1.14 (0.93)	.02	0.20
Sleep duration	0.42 (0.71)	0.46 (0.75)	0.37 (0.66)	.13	0.14
Habitual sleep efficiency	0.38 (0.78)	0.45 (0.86)	0.30 (0.67)	.03	0.19
Sleep disturbances	1.06 (0.53)	1.12 (0.53)	1.00 (0.53)	.009	0.23
Sleeping medication use	0.17 (0.59)	0.21 (0.66)	0.12 (0.50)	.06	0.17
Daytime dysfunction	1.53 (0.98)	1.68 (0.97)	1.37 (0.96)	<.001	0.32
Total score	5.92 (3.29)	6.48 (3.32)	5.33 (3.16)	<.001	0.36
**Social support (SSRS** ^**d**^ **), n (%)**					
Low level (total score ≤22)	70 (13.8)	56 (21.6)	14 (5.6)	<.001	36.42 (2)
Medium level (total score 23-44)	415 (81.5)	199 (76.8)	216 (86.4)		
High level (total score >45)	24 (4.7)	4 (1.6)	20 (8)		
**Social support (SSRS), mean (SD)**					
Objective support	7.55 (2.34)	7.23 (2.35)	7.89 (2.28)	.001	−0.28
Subjective support	16.17 (5.59)	14.54 (5.09)	17.85 (5.60)	<.001	−0.62
Utilization of support	7.49 (1.97)	7.04 (1.88)	7.96 (1.95)	<.001	−0.48
Total score	31.21 (7.96)	28.81 (7.41)	33.70 (7.75)	<.001	−0.65

a SMW: sexual minority women.

b CHW: cisgender heterosexual women.

c PSQI: Pittsburgh Sleep Quality Index.

d SSRS: Social Support Rating Scale.

### Associations Between Social-Ecological Factors and Sleep

The correlations among sleep quality (PSQI total score and seven component scores) and social support (SSRS total score and three domain scores), social relationship Qol, and environment Qol are reported in Table 3. The total sleep quality was significantly negatively associated with social support and all domains with weak correlations (*r* ranged from −0.15 to −0.26, *P*<.001), and it was significantly negatively linked with social relationship Qol (*r*=−0.38, *P*<.001) and environment Qol (*r*=−0.39, *P*<.001) with moderate correlations.

**Table 3.** Correlations between sleep quality, social support, and quality of life.

Sleep quality (PSQI^a^)	Social support (SSRS^b^)				Quality of life	
Objective	Subjective	Utilization	Sum score	Social	Environment
**Subjective sleep quality**						
*r*	−0.07	−0.17	−0.13	−0.17	−0.23	−0.26
*P* value	.14	<.001	.005	<.001	<.001	<.001
**Sleep latency**						
*r*	−0.13	−0.12	−0.16	−0.16	−0.21	−0.19
*P* value	.004	.005	<.001	<.001	<.001	<.001
**Sleep duration**						
*r*	−0.12	−0.15	−0.13	−0.17	−0.26	−0.28
*P* value	.007	<.001	.003	<.001	<.001	<.001
**Habitual sleep efficiency**						
*r*	−0.12	−0.15	−0.18	−0.18	−0.25	−0.26
*P* value	.007	<.001	<.001	<.001	<.001	<.001
**Sleep disturbances**						
*r*	−0.09	−0.12	−0.13	−0.14	−0.26	−0.29
*P* value	.04	.009	.003	.001	<.001	<.001
**Sleeping medication use**						
*r*	−0.12	−0.12	−0.09	−0.14	−0.10	−0.15
*P* value	.007	.007	.04	.001	.02	<.001
**Daytime dysfunction**						
*r*	−0.02	−0.18	−0.08	−0.15	−0.32	−0.25
*P* value	.66	<.001	.09	<.001	<.001	<.001
**Total score**						
*r*	−0.15	−0.24	−0.21	−0.26	−0.38	−0.39
*P* value	<.001	<.001	<.001	<.001	<.001	<.001

a PSQI: Pittsburgh Sleep Quality Index.

b SSRS: Social Support Rating Scale.

Comparisons of overall sleep quality between different socioecological groups are shown in Table S1 in Multimedia Appendix 1, with significant differences in sleep quality between people with different sexual identities, smoking status, alcohol use status, number of friends, and perceived social support (all *P*<.01). Table S2 in Multimedia Appendix 1 presents the multiple linear regression model of all the significant variables on sleep quality, and SEM was then performed among all these significant variables.

### Model Testing

**Figure 2.**
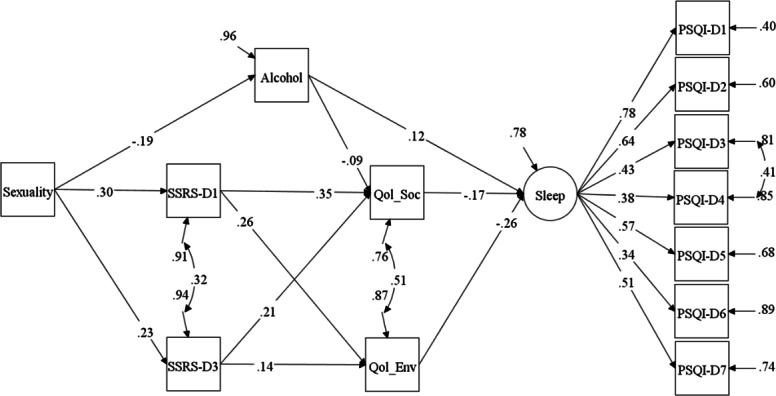
Final structural equation model of this study. Correlations and paths with nonsigniﬁcant *P* values are not depicted. PSQI: Pittsburgh Sleep Quality Index; PSQI-D1: subjective sleep quality; PSQI-D2: sleep latency; PSQI-D3: sleep duration; PSQI-D4: habitual sleep efficiency; PSQI-D5: sleep disturbance; PSQI-D6: sleeping medication use; PSQI-D7: daytime dysfunction; Qol: quality of life; Qol_Soc: social relationship of Qol; Qol_Env: environment domain of Qol; SSRS: Social Support Rating Scale; SSRS-D1: objective support; SSRS-D3: utilization of support.

The full initial model including smoking and number of friends failed because these 2 variables were neither directly nor indirectly correlated to sleep in the model and were therefore not included in subsequent model analyses. Contrary to the hypothesized model, none of the aspects of social support were found to have a direct relationship with sleep quality. Furthermore, the model involving the subjective support domain of social support (SSRS-D2) showed no direct or indirect association of this domain with sleep and it was therefore also removed. The final model was then proposed and illustrated in Figure 2, exhibiting robust fit indices (*χ*^2^_51_=118.80; RMSEA=0.051, 90% CI 0.039-0.063; CFI=0.948, TLI=0.921, SRMR=0.040).

All model standardized coefﬁcients (β), their standard errors, and associated *P* values are reported in Table 4. There were 6 significant paths from sexual identity (sexuality) to sleep quality in the final SEM model. Specifically, alcohol use (*β*=0.12, *P*=.01), social relationship Qol (*β*=−0.17, *P*=.004), and environment Qol (*β*=−0.26, *P*<.001) were direct predictors of sleep quality. Although social support did not directly inﬂuence sleep, it may indirectly influence sleep by aﬀecting the perceived social relationship Qol and environment Qol (*β* range 0.14‐0.35, *P*<.01).

**Table 4.** Standardized path coefficients and standard errors for all pathways in the final model.

Pathways	β^a^	SE	*P* value
Sexuality → Sleep quality	−0.07	0.05	.17
Sexuality → Alcohol use	−0.19	0.04	<.001
Sexuality → SSRS-D1^b^	0.30	0.04	<.001
Sexuality → SSRS-D3^c^	0.23	0.04	<.001
Sexuality → Qol_Soc^d^	0.03	0.04	.51
Sexuality → Qol_Env^e^	0.05	0.04	.23
Alcohol use → Sleep quality	0.12	0.05	.01
Alcohol use → Qol_Soc	−0.09	0.04	.02
Alcohol use → Qol_Env	0.00	0.04	.98
SSRS-D1 → Sleep quality	−0.03	0.05	.65
SSRS-D1 → Qol_Soc	0.35	0.04	<.001
SSRS-D1 → Qol_Env	0.26	0.05	<.001
SSRS-D3 → Sleep quality	−0.06	0.05	.25
SSRS-D3 → Qol_Soc	0.21	0.04	<.001
SSRS-D3 → Qol_Env	0.14	0.05	.002
Qol_Soc → Sleep quality	−0.17	0.06	.004
Qol_Env → Sleep quality	−0.26	0.06	<.001

a β: standardized coefﬁcient.

b SSRS-D1: objective support domain of social support.

c SSRS-D3: utilization of support domain of social support.

d Qol_Soc: social relationship domain of quality of life.

e Qol_Env: environment domain of quality of life.

## Discussion

### Principal Findings

This study was the first to explore the social-ecological pathways from sexual identity to sleep quality. Few studies worldwide have examined the sleep health of SMW, and this investigation was the first to examine the sleep health of Chinese women with diverse sexual identities. This study showed SMW reported significantly worse overall sleep quality relative to CHW, which is consistent with existing reviews [14-16] and findings among Chinese youth [24,25]. Compared with the pooled PSQI mean score of the general Chinese population from a recent meta-analysis [72], our study populations reported higher total scores on the same standardized PSQI scale (5.92 vs 4.32), implying that the female population in this study experienced noticeably poorer sleep quality. Furthermore, compared with the percentage of Korean sexual minority adults (lesbian, gay, and bisexual) reporting poor sleep quality (33.8%) [73], the rate of SMW reporting poor sleep quality in our study (55.2%) was higher, indicating that sleep in SMW requires more attention and improvement.

This study also found that the proportions of current smokers and alcohol users were significantly higher among SMW than those among CHW, which is consistent with findings in China and overseas [28,74]. A scoping review summarized that some Chinese SMW regard smoking and drinking as a means of interacting with other SMW in specific social gatherings. It is also reported that smoking and drinking were the main strategies they used as a sexual minority to cope with marital stress and feeling isolated [28]. These also echo our findings on the mental health of this sample population, where we have found that SMW indeed significantly experienced more psychological symptoms [56]. A national survey conducted in the United States revealed higher rates of substance use disorders among sexual minority adults compared to heterosexual adults, with SMW showing the highest rates [75], suggesting that substance use among women, especially SMW, needs to be given attention as a public health issue.

Alcohol is one of the most commonly used psychoactive substances in different social communities around the world. A growing body of literature has confirmed that alcohol use has deleterious effects on sleep by reducing sleep duration and sleep quality [35,36], which has also been documented in sexual minority men [76] and is consistent with this study. However, a meta-analysis of cohort studies indicates that the relationship between alcohol consumption and sleep disorders is conditional, with pooled analyses finding that general drinking and the incidence of sleep disorder were significantly correlated, while heavy drinking was not [37]. Another review concluded that alcohol use has a bidirectional relationship with sleep continuity disturbance [77]. For instance, insomnia symptoms were associated with subsequent heavy drinking; conversely, heavy drinking was associated with subsequent insomnia symptoms [78]. Nevertheless, both comparative analyses and the final SEM in this study revealed that drinking alcohol was significantly associated with poor sleep quality compared to not drinking alcohol, suggesting that both alcohol use and sleep in women deserve more attention and tailored intervention. Considering the limitations of the cross-sectional design used in this study, which could not provide evidence of causal relationships between drinking and sleep quality, more longitudinal studies with further subdivision of alcohol use are warranted in the future.

The adverse effects of smoking on sleep are well documented, as evidenced by the fact that smokers are more likely to experience sleeping difficulties, longer sleep latency, and poorer sleep quality [38,39,79], and similar findings were observed in the all-female population included in this study. The possible mechanism regarding the effects of smoking on sleep may be that smoking triggers depression or sleep-related breathing problems, which can lead to poor sleep quality [79]. However, a meta-analysis that included only cohort studies showed that, despite the significant correlation between smoking and the prevalence of insomnia, the difference between smokers and nonsmokers was very small (odds ratio 1.07) [80]. This may partly explain why the smoking variable that differed in between-group comparisons in our study was no longer associated with sleep quality in our final SEM. Future studies should record and subdivide smoking status and use prospective study designs to further explore the relationship between smoking and sleep across sexual identities.

The correlation between social support and sleep has been extensively studied in different populations [42,43,45] but its exploration in sexual minorities remains scarce. Therefore, the strength of this study is to fill this gap by examining the association between social support and sleep quality among Chinese women with diverse sexual identities. Consistent with previous research [49], overall social support was significantly and negatively associated with each component of sleep quality in the current study sample. Moreover, sexual minority individuals generally experience a higher level of poor social relationships, especially in the relatively traditional Chinese culture, where such groups are often invisible and therefore perceive more discrimination and stress [27,28,56]. However, findings from a study of 3 generations showed that feeling stigma was statistically significant in predicting a sleep disorder diagnosis among sexual minority individuals [81].

A mediation analysis of Chinese college students nationwide found that the relationship between sexual orientation and sleep quality was independently and in series mediated by interpersonal relationships and depressive symptoms, and this effect was more robust in men than women [25]. These interpersonal difficulties were related to their perceived stress associated with sleep difficulties. Our findings align with previous studies that found that sexual minority status is significantly associated with poor sleep quality [23], and social support has both direct and indirect correlations with sleep quality [42]. The relationships manifested in this study were that sexual identity was significantly associated with inadequate levels of social support, which in turn was significantly associated with poor levels of quality of life in terms of social relationships and environment, all of which were associated with poor sleep quality. Therefore, the lack of social support for sexual minority women is a significant public health issue, as is their poor sleep health, which deserves more attention.

Our correlation findings suggest that there may be more complex pathways from sexual identity to sleep among Chinese women with different sexual identities. Therefore, based on the holistic perspective proposed by the social-ecological model of sleep [32], this study explored and demonstrated the socioecological pathways from sexual identity to sleep quality for the first time using the multivariate SEM analysis method, providing a comprehensive evidence reference for promoting sleep health. The results of the final fitted model with satisfactory fit indices showed that starting from sexual identity, the following factors played important roles in women’s sleep quality: alcohol use; objective social support and utilization of support; and social relationship and environment Qol. Our findings demonstrated the important roles of socioecological factors on sleep quality among women with different sexual identities. Therefore, future research should consider not only modifiable individual-level factors but also the potential influence of social- and societal-level factors when developing and implementing sleep promotion interventions. In particular, future interventions targeting sleep quality in sexual minority women may benefit from incorporating strategies that improve their social support, alcohol use, and the quality of their living environment.

This study has several limitations. First, due to the cross-sectional design, it was not possible to determine the explicit causal relationships among variables, so further research using longitudinal designs is needed. Second, despite the diversity of sexual identities, our sample was an all-female population (self-identiﬁed as female or assigned female at birth), so the results might not be generalizable to the broader Chinese sexual minority population. There has also been research indicating that bisexual people [82], those with less education [83], and those who are economically disadvantaged [16] may be more likely to suffer from sleep disorders than other groups. Hence, future studies with more diverse and representative samples including individuals from varied socioeconomic backgrounds are warranted. Third, the social-ecological model of sleep used in this study is only the upstream end of sleep research, and the downstream effects, namely the effects of sleep on specific health outcomes, have not been expanded upon in this study. Therefore, further research is recommended to comprehensively explore sleep and holistic health across diverse populations.

### Conclusion

Overall, this study contributes to the existing literature by including SMW and expanding knowledge on sleep health in this population. By exploring socioecological pathways from sexual identity to sleep quality, this study provides a comprehensive understanding of factors that affect sleep health at different levels. These contextual and modifiable factors, based on the socioecological model of sleep, are layered and may interact with one another, providing guidance for future interventions. In addition, the pathways from sexual identity to sleep identified in this study are complex and multidisciplinary; thus, all stakeholders and sleep professionals across disciplines are encouraged to collaborate and contribute to sleep improvement programs tailored for sexual minority individuals.

## Supplementary material

10.2196/53549 Multimedia Appendix 1 Table S1: Sleep quality of the study sample with comparisons; Table S2: Multiple linear regressions of sleep quality.

## Abbreviations

CFI: comparative fit index
CHW: cisgender heterosexual women
PSQI: Pittsburgh Sleep Quality Index
Qol: quality of life
Qol_Env: environment domain of quality of life
Qol_Soc: social relationship domain of quality of life
RMSEA: root mean square error of approximation
SEM: structural equation modeling
SMW: sexual minority women
SRMR: standardized root mean square residual
SSRS: Social Support Rating Scale
TLI: Tucker-Lewis index
WHOQOL-BREF: World Health Organization Quality of Life–abbreviated short version
