# Supplementary material for: Exploring Social-Ecological Pathways From Sexual Identity to Sleep Among Chinese Women: Structural Equation Modeling Analysis
Source: JMIR Public Health Surveill. 2025 Jan 21;11:e53549. doi: 10.2196/53549 (PMC11774323; doi:10.2196/53549)
Supplement: Multimedia Appendix 1 [file publichealth-v11-e53549-s001.docx]

# Table S1 Sleep quality of the study sample with comparisons

| Variables | | PSQI^a^  mean(SD) | F/t^b^ | *P* value |
| --- | --- | --- | --- | --- |
| **Sexual identity** | | | | |
|  | Cisgender heterosexual women | 5.33(3.16) | 4.014 | <.001 |
|  | Sexual minority women | 6.48(3.32) |  |  |
| **Age group** | | | | |
|  | 18-20 | 5.85(3.18) | 1.503 | .37 |
|  | 21-30 | 5.84(3.30) |  |  |
|  | 31-40 | 6.61(3.55) |  |  |
|  | 41-56 | 5.25(2.67) |  |  |
| **Smoking** | | | | |
|  | Never smoked or have quitted | 5.71(3.19) | -3.225 | .001 |
|  | Current smoker | 6.99(3.63) |  |  |
| **Alcohol use** | | | | |
|  | Never drank or have quitted | 5.25(3.39) | -3.393 | .001 |
|  | Current alcohol user | 6.27(3.19) |  |  |
| **Drug use** | | | | |
|  | Never used before | 5.92(3.31) | 0.024 | .98 |
|  | Have used drug before | 5.89(2.26) |  |  |
| **Ethnicity** | | | | |
|  | Han people | 5.87(3.23) | -0.999 | .32 |
|  | Others (Muslim, et al.) | 6.40(3.92) |  |  |
| **Education, n(%); *P*<.001** | | | | |
|  | High school and below | 6.55(3.84) | 2.489 | .08 |
|  | College/ Bachelor | 6.11(3.33) |  |  |
|  | Graduate degree and above | 5.52(3.10) |  |  |
| **Monthly income (China Yuan)** | | | | |
|  | ≤1000 | 6.14(3.07) | 1.380 | .22 |
|  | 1001-3000 | 6.18(3.19) |  |  |
|  | 3001-5000 | 6.30(3.79) |  |  |
|  | 5001-7000 | 6.82(3.68) |  |  |
|  | 7001-9000 | 5.13(3.41) |  |  |
|  | 9001-11000 | 5.93(3.28) |  |  |
|  | More than 11000 | 5.59(3.16) |  |  |
| **Number of friends** | | | | |
|  | 0 | 5.46(3.08) | 3.027 | .006 |
|  | 1-5 | 6.20(3.23) |  |  |
|  | 6-10 | 5.85(3.76) |  |  |
|  | 11-20 | 6.81(3.52) |  |  |
|  | 21-50 | 7.00(3.43) |  |  |
|  | 51-100 | 5.33(2.84) |  |  |
|  | >100 | 4.44(2.39) |  |  |
| **Relationship** | | | | |
|  | Have a steady partner | 6.00(4.00) | 0.506 | .61 |
|  | Have no steady partner(s) | 6.00(3.00) |  |  |
| **Bed sharing status** | | | | |
|  | Separate bed in separate room | 6.03(3.49) | 1.388 | .25 |
|  | Separate bed in shared room | 6.17(3.33) |  |  |
|  | Sharing same bed with partner | 5.53(2.81) |  |  |
| **Local resident or not** | | | | |
|  | Local resident | 5.89(3.50) | -0.132 | .90 |
|  | Non-local resident (Migrant) | 5.93(3.18) |  |  |
| **Duration in current residence** | | | | |
|  | <3 months | 5.90(3.60) | 0.464 | .71 |
|  | 3-6 months | 5.47(3.99) |  |  |
|  | 7-12 months | 5.36(3.43) |  |  |
|  | >1 year | 5.98(3.22) |  |  |
| **Cohabitation status** | | | | |
|  | Live alone | 6.18(3.40) | 0.741 | .59 |
|  | Live with same-sex partner | 6.00(3.23) |  |  |
|  | Live with opposite-sex partner | 5.32(2.89) |  |  |
|  | Live with friends | 6.08(3.45) |  |  |
|  | Live with families | 5.85(3.29) |  |  |
|  | Others | 6.19(3.53) |  |  |
| **Social support** | | | | |
|  | Low level | 7.57(3.45) | 15.525 | <.001 |
|  | Medium level | 5.76(3.20) |  |  |
|  | High level | 3.71(2.29) |  |  |

^a^PSQI: Pittsburgh Sleep Quality Index total score.

^b^F/t: The coefficients of one-way ANOVA (F value) or independent *t*-tests (t value).

# Table S2 Multiple linear regressions of sleep quality (PSQI^a^ total score)

|  | Coeff^b^ | *P* value | 95% CI^c^ |
| --- | --- | --- | --- |
|  |  |  |  |
| Sexual majority (vs. Sexual minority) | -0.22 | .48 | (-0.81,0.38) |
| Alcohol user (vs. never before or has quit) | 0.64 | .03 | (0.07,1.21) |
| Current smoker (vs. never before or has quit) | 0.08 | .83 | (-0.69,0.86) |
| Social relationship domain of Qol^d^ | -0.20 | <.001 | (-0.31,-0.09) |
| Environment domain of Qol | -0.30 | <.001 | (-0.42,-0.18) |
| Objective support (SSRS-D1^e^) | -0.02 | .61 | (-0.07,0.04) |
| Subjective support (SSRS-D2^f^) | 0.01 | .91 | (-0.12,0.13) |
| Utilization of support (SSRS-D3^g^) | -0.08 | .27 | (-0.23,0.07) |
| Number of friends (1-5) | 0.32 | .36 | (-0.37,1.01) |
| Number of friends (6-10) | 0.42 | .33 | (-0.43,1.28) |
| Number of friends (11-20) | 1.05 | .05 | (0.03,2.07) |
| Number of friends (21-50) | 1.39 | .01 | (0.34,2.43) |
| Number of friends (51-100) | 0.04 | .95 | (-1.19,1.27) |
| Number of friends (>100) | -0.74 | .24 | (-1.97,0.50) |
| *R-square* | *22.8%* | | |

^a^PSQI: Pittsburgh Sleep Quality Index.

^b^Coeff: Coefﬁcient.

^c^CI: Conﬁdence Interval.

^d^Qol: Quality of life.

^e^SSRS-D1: Objective support domain of social support.

^f^SSRS-D2: Subjective support domain of social support.

^g^SSRS-D3: Utilization of support domain of social support.
